# Syndromic management of sexually transmitted infections in the Brazilian Amazon: A 10-year retrospective study

**DOI:** 10.1371/journal.pntd.0014282

**Published:** 2026-05-04

**Authors:** Ana Claudia Chaves Camilo, Valderiza Lourenço Pedrosa, José Carlos Sardinha, Silvana Paiva da Costa, Celília de Lima Borges, Angelica Espinosa Miranda, Adriana Bindá, André Luiz Leturiondo, Camila Gurgel dos Santos Silva, Cynthia de Oliveira Ferreira, Maria das Graças Barbosa Guerra, Sinesio Talhari, Carolina Talhari

**Affiliations:** 1 Programa de Pós-Graduação em Ciências Aplicadas à Dermatologia, Universidade do Estado do Amazonas, Manaus, Amazonas, Brazil; 2 Fundação Hospitalar Alfredo da Matta de Dermatologia, Manaus, Amazonas, Brazil; 3 Programa de Pós-Graduação em Doenças Infecciosas, Universidade Federal do Espirito Santo, Manaus, Brasil; University of Connecticut College of Agriculture Health and Natural Resources, UNITED STATES OF AMERICA

## Abstract

**Background:**

The syndromic approach is a widely applied strategy for managing sexually transmitted infections (STIs) in settings where laboratory diagnostics are limited. This study aimed to describe temporal patterns in syndromic STI diagnoses and identify factors independently associated with these diagnoses over a period of 10 years at a reference center in the state of Amazonas, Brazilian Amazon.

**Methodology/Principal findings:**

A retrospective review was conducted using medical records from patients attending a reference center for STI care in Amazonas, Brazil, from 2014 to 2023. Sociodemographic, clinical, and behavioral data were analyzed. The syndromic classification followed World Health Organization guidelines and included vaginal discharge, cervical discharge, urethral discharge, genital ulcers, and genital warts. A total of 32,485 medical records were reviewed, with 14,931 (46.0%) syndromic diagnoses identified. The most frequent syndromes were genital warts (41.0%), urethral discharge (31.5%), genital ulcer (21.3%), cervical discharge (3.8%), and vaginal discharge (2.3%). In multivariate analysis, factors independently associated with receiving a syndromic diagnosis were age ≥ 30 years (OR = 1.16; 95% CI: 1.05–1.20), male sex (OR = 2.31; 95% CI: 1.75–2.45), single marital status (OR = 1.82; 95% CI: 1.43–2.21), irregular condom use (OR = 3.21; 95% CI: 2.46–3.60), and having two or more casual partners (OR = 3.42; 95% CI: 2.94–3.90).

**Conclusions/Significance:**

Despite inherent limitations, the syndromic approach remains an essential strategy for STI control in resource-constrained contexts such as the Amazon. It facilitates prompt treatment and broadens access to care where laboratory confirmation is unavailable. Integrating syndromic and etiological approaches is vital to improve diagnostic accuracy, optimize antimicrobial use, and strengthen public health responses to STIs in the region.

## Author summary

Sexually transmitted infections remain a major public health challenge, especially in areas where access to laboratory tests is limited. In the Amazon region of Brazil, the vast geography and the difficulty in reaching many communities make timely diagnosis and treatment even harder. In this study, we analyzed data from more than 32,000 patients seen at a referral center in Manaus over the past ten years. Using the syndromic approach—a strategy that guides treatment based on signs and symptoms rather than lab tests—we identified the main infection patterns and the groups most affected. We found that men, people over 30 years old, those who were single, and individuals with irregular condom use or multiple partners were more likely to receive a syndromic diagnosis. Our results highlight how this approach remains essential to ensure access to care in resource-limited settings, while also reinforcing the need to expand laboratory testing to improve accuracy and guide better treatment strategies.

## Introduction

Sexually transmitted infections (STIs) remain a major global public health challenge, particularly in low- and middle-income settings where timely access to laboratory diagnostics is still limited [1,2]. To address these constraints and other barriers to effective STI care, including delayed treatment, loss to follow-up, and stigma-related healthcare avoidance, the World Health Organization (WHO), promoted syndromic management, which relies on standardized clinical algorithms based on specific clinical signs and symptoms to guide same-visit treatment at the initial consultation, an important advantage in settings where patients may not return for test results, without etiological confirmation [1–3].

In recent decades, advances in diagnostic technologies, including rapid tests and molecular methods, have expanded the feasibility of more precise identification of specific pathogens, supporting an etiological approach to STI management [ 4–7]. However, the implementation of laboratory-based strategies remains operationally complex in many real-world settings, requiring sustained infrastructure, supply chains, and trained personnel. As a result, syndromic management continues to be widely used and remains highly relevant in regions where laboratory diagnoses are unavailable, inaccessible or inconsistently implemented [1,4,8].

In the Northern region of Brazil, STI control faces substantial challenges due to geographic, socioeconomic, and structural factors. The vast territory, with isolated and hard-to-reach communities, hampers the implementation of prevention, testing, and treatment strategies. Additionally, unequal access to healthcare services, low coverage of primary care in certain areas, and a shortage of trained professionals for STI management further exacerbate the situation. Sociocultural issues, such as taboos surrounding sexuality and educational barriers, also limit the demand for diagnosis and adherence to treatment [9–12]. Overcoming these challenges requires strengthening primary healthcare, expanding access to rapid testing and telemedicine strategies, and promoting educational and awareness-raising initiatives targeting the most vulnerable populations.

The syndromic approach continues to be valuable in STI management, particularly within primary healthcare settings, and should be viewed as complementary to etiologic diagnosis [1,3]. Furthermore, examining the public health implications of its continued use, such as the management of vulnerable populations, resource optimization, and the reduction of complications associated with delayed treatment, is an important strategy. Despite its widespread use, long-term, service-based evaluations of syndromic STI management in the Brazilian Amazon remain scarce, and the available evidence is largely limited to short-term studies or incomplete population-based surveillance.

Globally, syndromic STI algorithms enable prompt treatment where etiologic testing is limited, but they have recognized limitations, including suboptimal specificity, overtreatment, and antimicrobial stewardship concerns. Recent guidance therefore recommends reducing exclusive reliance on syndromic management and integrating rapid tests and molecular diagnostics where feasible [1,4]. In Brazil, syndromic management remains recommended in primary care, and service-based experience indicates that combining syndromic assessment with rapid testing, particularly for HIV and syphilis, can strengthen case management and linkage to care within the SUS. In the Brazilian Amazon, evaluations remain comparatively sparse and often short term, supporting the need for longitudinal, service-based analyses in geographically constrained settings [9–21].

To address this gap, particularly the scarcity of long-term, service-based evaluation of syndromic STI management in the Brazilian Amazon, where geographic and structural barriers can delay etiologic testing and compromise follow-up, this study aimed to describe temporal patterns in syndromic STI diagnoses and to identify factors independently associated with these diagnoses over a 10-year period in a referral center in the state of Amazonas, Brazilian Amazon. By examining the historical performance and public health relevance of this approach in a real-world referral setting, we aim to highlight its strengths, limitations, and implications for future integration with etiologic strategies.

## Methods

### Ethics statement

This study was conducted as a part of a broader research project entitled “Clinical and Laboratory Management of Sexually Transmitted Infections in a Referral Center in Amazonas”. The project was approved by the Research Ethics Committee of FUHAM (protocol CAAE 83020224.10000.0002). The requirement for informed consent was waived because the study anonymized secondary data from medical records, in accordance with national ethical regulations and Resolution 466/12 of the Brazilian National Health Council.

### Study design and setting

**Fig 1:**
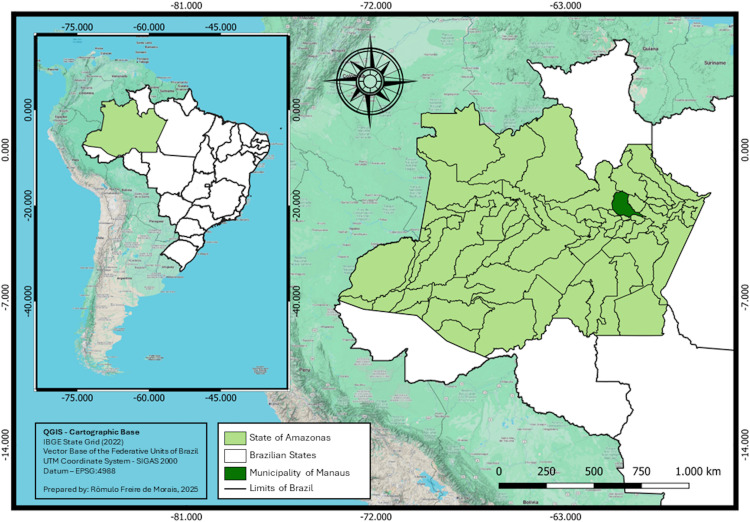
Geographic location of Manaus within the State of Amazonas and in the national context of Brazil. Map created in QGIS using IBGE (Instituto Brasileiro de Geografia e Estatística) administrative boundary shapefiles (https://www.ibge.gov.br/geociencias/organizacao-do-territorio/malhas-territoriais/15774-malhas-territoriais.html) as the base layer.

This retrospective observational study consisted of a comprehensive review of medical records of patients attended at the Alfredo da Matta Tropical Dermatology and Venereology Foundation (AMTDV) between January 2014 and December 2023. AMTDV is a state-level referral center for Tropical Dermatology and STI, operating under the State Health Secretariat of Amazonas. The institution is located in Manaus, the capital of Amazonas, which serves as a major financial, commercial, and touristic hub in Northern Brazil and attracts patients from both urban and remote of the state (Fig 1).

### Study population

The study population included all individuals who attended AMTDV during the study period, regardless of the reason for consultation. Medical records were systematically reviewed to identify visits with available sociodemographic and clinical information. Records were included in the analysis if they contained sufficient data to ascertain the presence or absence of a syndromic STI diagnosis. Records with missing or inconsistent key variables that precluded ascertainment of the outcome were excluded.

### Data collection

Data were extracted retrospectively from the electronic medical record system using a standardized data abstraction instrument developed for this study. Extracted variables included:

(a) Sociodemographic characteristics: age, sex, race/ethnicity (skin color), marital status, education level and sexual orientation.
(b) Clinical characteristics: reported signs and symptoms at presentation, self-reported history of previous STI, and type of consultation (spontaneous demand or referral).
(c) Diagnostic information: clinical syndromic classification according to the WHO syndromic management approach [1], including vaginal discharge, cervical discharge, genital ulcer, urethral discharge, and genital warts.

### Outcome variable

The primary outcome (dependent variable) was the presence of a syndromic STI diagnosis (yes/no), defined according to WHO guidelines for syndromic management criteria. This outcome reflects a clinical classification among individuals attending a referral service and should not be interpreted as population-level STI prevalence.

### Independent variables

Independent variables included age (analyzed both as a continuous and categorized), sex, race/ethnicity, marital status, education level, sexual orientation, number of casual partners, number of steady partners, history of previous STIs, HIV and syphilis screening, year of diagnosis, and type of consultation.

### Statistical analysis

Descriptive statistics were used to characterize the study population and to describe temporal patterns in syndromic STI diagnoses s over the study period. Absolute and relative frequencies were calculated for categorical variables and means (± standard deviations) were calculated for continuous variables, depending on distribution.

The unit of analysis for regression models was the syndromic diagnosis case, defined as a recorded clinical syndrome at a given encounter. Because some individuals contributed more than one syndromic diagnosis during the study period, either across different visits or, less frequently, within the same visit, observations were not assumed to be independent.

Associations between independent variables and the presence of a syndromic STI diagnosis were assessed using logistic regression models with standard errors clustered at the patient level to account for within-individual correlation. Variables with a p-value <0.20 in univariate analyses were considered for inclusion in the multivariable model. A backward stepwise selection procedure was used to derive a set of variables independently associated with the outcome. Adjusted odds ratios (OR) with 95% confidence intervals (CI) were reported.

Multivariable analyses were conducted using a complete-case approach. No imputation was performed because missing data primarily reflected incomplete routine clinical documentation and were considered unlikely to be missing at random. The number of syndrome cases excluded from each model due to missing data is reported.

Model fit was assessed using the Hosmer-Lemeshow goodness-of-fit test, and multicollinearity was evaluated using variance inflation factors. Statistical significance was defined as a two-sided p-value <0.05.

## Results

A total of 32,485 individuals sought care at the referral center during the study period, of whom 69.4% presented through spontaneous demand. Overall, 14,931 (46,0%) syndromic diagnoses were recorded in 14,513 individuals, as some patients presented with more than one syndrome during the visit.

The most frequent diagnosed syndromes were genital warts (6,128 cases; 41.0%), followed by urethral discharge (4,710; 31.5%), genital ulcer (3,185; 21.3%), cervical discharge (571; 3.8%), and vaginal discharge (337; 2.3%) (Table 1).

**Table 1:** Historical series of syndromic diagnoses recorded at a referral STI center in Amazonas, Brazil, from 2014 to 2023.

Syndrome	2014	2015	2016	2017	2018	2019	2020	2021	2022	2023	Total
**Vaginal discharge**	41	34	58	31	15	34	17	27	28	52	337
**Cervical discharge**	131	78	108	104	57	45	10	17	6	15	571
**Genital ulcer**	521	528	519	478	299	282	65	83	247	163	3.185
**Urethral discharge**	876	753	694	628	437	408	240	72	279	323	4.710
**Genital warts**	764	807	907	760	673	572	301	620	400	324	6.128
**TOTAL**	2.333	2.200	2.286	2.001	1.481	1.341	633	819	960	877	14.931

**Fig 2:**
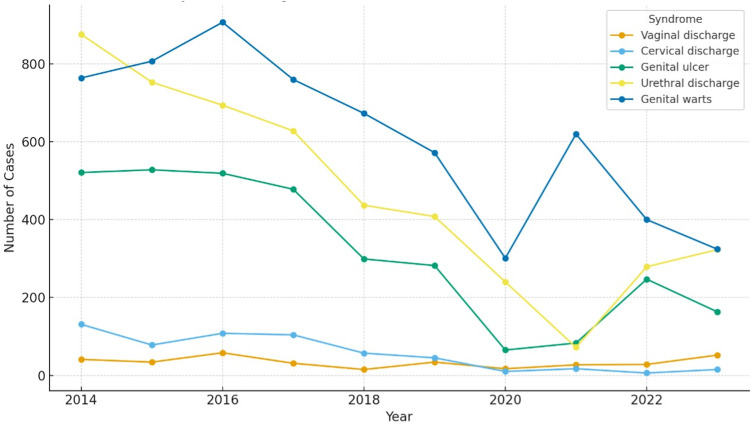
Temporal trends in syndromic STI diagnoses recorded at a referral STI center in Amazonas, Brazil, 2014–2023.

Temporal trends in syndromic STI diagnoses over the 10-year study period are illustrated in Fig 2. Annual records decreased after 2017, with the lowest numbers observed in 2020 and 2021.

In addition, although not a primary outcome of the study, 22,160 individuals were tested for HIV and syphilis during routine care. Among those tested, 1,685 (7.6%) were HIV-positive, and 4,363 (19.7%) tested positive for syphilis, highlighting a substantial burden of infection among individuals accessing syndromic STI services.

Table 2 shows the distribution of syndromic diagnoses (genital warts, urethral discharge, genital ulcer, cervical discharge, vaginal discharge) stratified by sex over the study period. The syndromes were more frequent among males than females (71.5% versus 28.5%).

**Table 2:** Distribution of syndromic diagnoses (genital warts, urethral discharge, genital ulcer, cervical discharge, vaginal discharge) stratified by sex, 2014-2023.

Syndrome/Sex	Male n (%)	Female n (%)	Total n (%)
**Vaginal discharge**	0 (0)	337 (100)	337 (2.3)
**Cervical discharge**	0 (0)	571 (100)	571 (3.8)
**Genital ulcer**	2.667 (83.7)	518 (16.3)	3.185 (21.3)
**Urethral discharge**	4.710 (100)	0 (0)	4.710 (31.5)
**Genital warts**	3.293 (53.7)	2.835 (46.3)	6.128 (41.1)
**TOTAL**	10.670 (71.5)	4.261 (28.5)	14.931 (100)

Sociodemographic characteristics showed that 64% were male, 57.6% were single, and 59.5% had 10 or more years of education. Nearly half of the population (48.6%) was aged 18–30 years (Table 3).

**Table 3:** Sociodemographic characteristics of syndromic diagnoses cases recorded at a referral STI center in Amazonas, Brazil, from 2014 to 2023.

Variables	Total	%	Syndromic diagnosis				P value
			Yes	%	No	%	
	32485		14513		17792		
**Age in years**							
18-30	18520	57.0	9317	50.3	9203	49.7	0.096
31-44	7971	24.5	3047	21.0	4924	27.7	< 0.005
45-59	4231	13.0	1268	8.7	2963	16.7	< 0.001
60 +	1763	5.4	481	3.3	1282	7.2	< 0.001
**Self-reported skin color/race**							
White	4514	13.9	1627	11.2	2887	16.2	< 0.001
Black	858	2.6	323	2.2	535	3.0	< 0.001
Asian	160	0.5	70	0.5	90	0.5	0.114
Mixed	22626	69.7	10762	74.2	11864	66.7	< 0.001
Indigenous	148	0.5	60	0.4	88	0.5	0.214
Not informed	4179	12.9	1671	11.5	2508	14.1	< 0.001
**Sex**							
Female	11690	36.0	3958	27.3	7732	43.5	< 0.001
Male	20795	64.0	10555	72.7	10240	57.6	0.2891
**Education**							
None	597	1.8	224	1.5	373	2.1	0.050
1 to 4 years	1982	6.1	671	4.6	1311	7.4	< 0.001
5 to 9 years	6283	19.3	3176	21.9	3107	17.5	0.384
High school	14532	44.7	6908	47.6	7624	42.9	0.004
College	4706	14.5	1649	11.4	3057	17.2	<0.001
Not informed	4385	13.5	1885	13.0	2500	14.1	< 0.001
**Marital status**							
Single	18706	57.6	9203	63.4	9503	53.4	0.283
Married/Living together	11022	34.0	4232	28.2	6790	38.2	< 0.001
Divorced	1065	3.3	317	2.2	748	4.2	< 0.001
Widow	334	1.0	71	0.5	263	1.5	< 0.001
Not informed	1358	4.2	690	4.8	668	3.8	0.551

Regarding sexual behavior, 41.5% reported first sexual intercourse at age 15 or younger, 55.1% reported one regular partner, 40.3% reported casual partners, and 29.2% reported never using condoms (Table 4).

**Table 4:** Behavioral characteristics of diagnoses cases recorded at a referral STI center in Amazonas, Brazil, from 2014 to 2023.

Variables	Total	%	Syndromic diagnosis				P value
			Yes	%	No	%	
	32485		14513		17792		
**Age at first sexual intercourse**							
≤ 15 years	13489	41.5	7158	49.3	6331	35.6	< 0.001
> 15 years	12174	37.5	5401	37.2	6773	38.1	0.005
Not informed	6822	21.0	1954	13.5	4868	27.4	< 0.001
**Regular partner**							
None	10621	32.7	5155	35.5	5466	30.7	0.255
1	17902	55.1	7734	53.3	10168	57.1	< 0.001
2-4	269	0.8	142	1.0	127	0.7	0.412
5 or more	40	0.1	18	0.1	22	0.1	0.879
Not informed	3653	11.2	1464	10.1	2189	12.3	0.248
**Casual partner**							
None	14777	45.5	5720	39.4	9057	50.9	< 0.001
1	6515	20.1	3532	24.3	2983	16.8	< 0.001
2-4	5111	15.8	2847	19.6	2264	12.8	0.186
5-9	1014	3.2	617	4.2	397	2.2	0.736
10 +	390	1.2	200	1.4	190	1.1	0.613
Not informed	4678	14.4	1597	11.0	3081	17.3	< 0.001
**Sexual practice**							
Homosexual	2678	8.2	715	4.9	1963	11.0	< 0.001
Bisexual	915	2.8	349	2.4	566	3.2	< 0.001
Heterosexual	21063	64.8	10357	71.4	10706	60.2	0.162
Not informed	7829	24.1	3092	21.3	4737	26.6	< 0.001
**Condom use**							
Always	3588	11.0	1304	9.0	2284	12.8	< 0.001
Sometimes	11733	36.1	6667	45.9	5066	28.5	< 0.001
Only with the regular partner	72	0.2	38	0.3	34	0.2	0.637
Only with casual partners	392	1.2	115	0.8	277	1.6	
Do not use	9473	29.2	3913	27.0	5560	31.3	< 0.001
Not informed	7227	22.2	2476	17.1	4751	26.7	< 0.001

In the multivariate logistic regression, factors independently associated with receiving a syndromic diagnosis included: age ≥ 30 years (OR = 1.16; 95% CI: 1.05–1.20), male sex (OR = 2.31; 95% CI: 1.75–2.45), single marital status (OR = 1.82; 95% CI: 1.43–2.21), irregular condom use (OR = 3.21; 95% CI: 2.46–3.60), and two or more casual partners (OR = 3.42; 95% CI: 2.94–3.90) (Table 5).

**Table 5:** Multivariate analysis of factors associated with syndromic diagnoses recorded at a referral STI center in Amazonas, Brazil, from 2014 to 2023.

Variables	OR	CI95%	p value
Age > 30 years vs. 18–30 years	1.16	1.05–1.20	0.0197
Male vs. female	2.31	1.75–2.45	0.0392
Single vs. married	1.82	1.43-2.21	0.0216
Higher education vs. basic education	2.64	1.92-3.39	0.0324
Irregular condom use vs. regular condom use	3.21	2.46–3.60	0.0185
Casual partners ≥ 2 vs. none	3.42	2.94-3.90	0.0137

## Discussion

This 10-year retrospective analysis of service-based from a referral center in the Brazilian Amazon documented a high frequency of syndromic STI diagnoses among individuals seeking care. The predominance of male patients is consistent with previous studies and may relate to differences in symptom presentation and healthcare utilization; however, motivations for attendance were not captured in this retrospective dataset [9,11,13–15]. A considerable proportion of attendees reported unprotected intercourse prior to presentation, highlighting the frequency of recent sexual risk exposure among individuals accessing care. In addition, the high frequency of casual sexual partnerships and and inconsistent condom use reflects persistent behavioral risk patterns [9–12,16,17]. These results are consistent with previous service-based studies from the Brazilian Amazon and other regions of Brazil, which have reported a high burden of syndromic STI diagnoses among sexually active populations, particularly men [9–12,16,17].

These findings are particularly relevant in the context of the Amazonas region, where vast geographic distances, limited health infrastructure, and the presence of riverside and indigenous populations pose structural challenges to STI prevention and care. In this setting, longitudinal data derived from a referral center provide a valuable opportunity to characterize service utilization and patient profiles over time, especially given the scarcity of population-based STI surveillance data in the region [10,11].

The predominance of urethral discharge and genital warts observed in this study reflects local clinical demand patterns and highlights the disproportionate burden of STI-related morbidity among men. These results are align with national surveillance data showing a sustained increase in syphilis and other STI in Brazil, particularly in the Northern region, where syphilis detection rates reached 93.7 cases/100,000 inhabitants in 2023 [15]. Differences in symptom presentation and healthcare-seeking behavior likely contribute to this pattern, as urethral discharge is typically more symptomatic and prompts care-seeking, whereas vaginal and cervical infections may be asymptomatic or perceived as less urgent. In addition, women may access STI-related care through other entry points, such as primary care, gynecological, or antenatal services [10], which may partly explain the lower frequency of vaginal and cervical discharge syndromes observed in this study.

The reduction in syndromic diagnoses observed during 2020–2021 likely reflects decreased healthcare utilization during the COVID-19 pandemic, as reported in other Brazilian health services, rather than a true decline in STI incidence [18].

In Amazonas, where many communities are accessible only by river or air transport, the capacity to provide immediate treatment without laboratory confirmation is particularly operationally important [10]. In such contexts, syndromic management continues to play a pragmatic role in bridging diagnostic gaps when access to laboratory-based testing is limited. At the same time, the limitations of syndromic management must be clearly acknowledged. Nonspecific clinical presentations may lead to misdiagnosis, overtreatment, and unnecessary antimicrobial exposure, with potential implications for increased antimicrobial resistance, especially in *Neisseria gonorrhoeae* [4,19]. For this reason, WHO has increasingly recommended reducing exclusive reliance on syndromic management and promoting integration with rapid tests and molecular diagnostics [1,4,20]. As a retrospective analysis of routinely collected service data, we report associations and cannot infer causality or the motivations underlying care-seeking.

In Brazil, recent national and international strategies have emphasized that combining syndromic assesment with rapid tests for HIV and syphilis can improve diagnostic accuracy, reduce unnecessary antibiotic use, and enhance linkage to care [8,21]. While the Ministry of Health continues to recommend syndromic management in primary care settings, national policies increasingly emphasize the integration of etiologic testing strategies within the SUS to strengthen STI surveillance and clinical management. Importantly, the behavioral and demographic factors independently associated with syndromic STI diagnoses in the multivariable analysis, particularly inconsistent condom use and multiple sexual partners, align with national surveillance data indicating sustained STI transmission in Brazil [3,22].

Several limitations should be considered when interpreting these findings. The use of routinely collected secondary data resulted in missing or incomplete information for some sociodemographic and behavioral variables, which may have biased subgroup analyses and limited adjustment for potential confounders. These limitations highlight the importance of strengthening the completeness and quality of health information systems, as accurate and comprehensive sociodemographic and clinical data are important to characterize patient profiles, identify inequities, and support more targeted and effective public health and clinical interventions. Despite these constraints, this study draws on data from one of the largest STI referral centers in Northern Brazil and provides valuable longitudinal overview of syndromic STI care in the Amazon. The findings offer insights relevant to service organization and policy development, including the potential value of mobile health strategies, community-based interventions, and expanded access to point-of-care and molecular diagnostics in remote settings.

From a programmatic perspective, these findings support prioritizing targeted risk-reduction and linkage strategies for groups more likely to receive a syndromic STI diagnosis in this service setting, including men, single individuals, and those reporting inconsistent condom use and multiple casual partners. Practical steps include strengthening same-day point-of-care testing integrated into syndromic consultations (particularly HIV and syphilis rapid tests where available), improving partner notification and treatment pathways, and expanding outreach models, such as mobile/river-based clinics and telehealth-supported follow-up, to mitigate geographic barriers and reduce loss to follow-up in remote communities [4,23].

Syndromic management remains a pragmatic and widely used strategy for STI care in geographically and resource-limited contexts such as the Amazon [24]. However, its greatest public health value lies in its integration with expanded diagnostic capacity, strengthened surveillance systems, and context-sensitive service delivery models. Adapting STI control strategies to the unique epidemiological and structural realities of the Amazon is important to improving equity, quality of care, and health outcomes.

This study provides a decade-long, service-based overview of syndromic STI diagnoses at a major referral center in the Brazilian Amazon, highlighting patterns of care-seeking and clinical presentation in a geographically and structurally constrained setting. The findings highlight the continued programmatic relevance of syndromic management for ensuring timely access to treatment, particularly where laboratory infrastructure is limited. At the same time, the results reinforce the need to integrate syndromic approaches with expanded diagnostic capacity, improved data completeness, and strengthened health information systems to enhance surveillance, guide targeted interventions, and reduce inequities in STI care. Tailoring STI control strategies to the epidemiological and operational realities of the Amazon remains important for improving quality of care and public health impact.

## References

[1] Wi TE, Ndowa FJ, Ferreyra C, Kelly-Cirino C, Taylor MM, Toskin I, et al. Diagnosing sexually transmitted infections in resource-constrained settings: challenges and ways forward. J Int AIDS Soc. 2019;22 Suppl 6(Suppl Suppl 6):e25343. doi: 10.1002/jia2.25343 31468679 PMC6715950

[2] World Health Organization. Global progress report on HIV, viral hepatitis and sexually transmitted infections, 2021[Internet]. Geneva: WHO; 2021 [cited 2025 Jan 31]. Available from: https://www.who.int/publications/i/item/9789240027077

[3] Brasil. Protocolo Clínico e Diretrizes Terapêuticas para Atenção Integral às Pessoas com Infecções Sexualmente Transmissíveis (IST) [Internet]. Brasília (DF): Ministério da Saúde; 2021 [cited 2025 May 25]. Available from: https://www.gov.br/aids/pt-br/central-de-conteudo/pcdts/2022/ist/pcdt-ist-2022_isbn-1.pdf/view

[4] World Health Organization. Guidelines for the management of symptomatic sexually transmitted infections[Internet]. Geneva: WHO; 2021 [cited 2025 Jan 31]. Available from: https://www.who.int/publications/i/item/9789240024168 34370424

[5] Bazzo ML, Machado HdM, Martins JM, Schörner MA, Buss K, Barazzetti FH, et al. Aetiological molecular identification of sexually transmitted infections that cause urethral discharge syndrome and genital ulcer disease in Brazilian men: a nationwide study. Sex Transm Infect. 2024;100(3):133–7. doi: 10.1136/sextrans-2023-055950 38360847

[6] de Souza LS, Sardinha JC, Talhari S, Heibel M, Santos MND, Talhari C. Main etiological agents identified in 170 men with urethritis attended at the Fundação Alfredo da Matta, Manaus, Amazonas, Brazil. An Bras Dermatol. 2021;96(2):176–83. doi: 10.1016/j.abd.2020.07.007 33640187 PMC8007485

[7] Mungati M, Machiha A, Mugurungi O, Tshimanga M, Kilmarx PH, Nyakura J, et al. The Etiology of Genital Ulcer Disease and Coinfections With Chlamydia trachomatis and Neisseria gonorrhoeae in Zimbabwe: Results From the Zimbabwe STI Etiology Study. Sex Transm Dis. 2018;45(1):61–8. doi: 10.1097/OLQ.0000000000000694 29240636 PMC5994235

[8] Gaspar PC, Barreto JOM, Bigolin A, Miranda AE, Aires Júnior LF, Bazzo ML, et al. Brazilian Clinical Practice Guidelines for Sexually Transmitted Infections That Cause Urethral Discharge: Assessment According to the AGREE II and Critical Analysis Based on the WHO Recommendations. Sex Transm Dis. 2023;50(12):804–9. doi: 10.1097/OLQ.0000000000001873 37824264

[9] Benzaken AS, Galbán Garcia E, Sardinha JC, Pedrosa VL, Paiva V. Intervenção de base comunitária para a prevenção das DST/Aids na região amazônica, Brasil. Rev Saude Publica. 2007;41(Suppl 2):118–26. doi: 10.1590/S0034-89102007000900018 18094795

[10] Garnelo L, Parente RCP, Puchiarelli MLR, Correia PC, Torres MV, Herkrath FJ. Barriers to access and organization of primary health care services for rural riverside populations in the Amazon. Int J Equity Health. 2020;19(1):54. doi: 10.1186/s12939-020-01171-x 32731874 PMC7394681

[11] Galvão JJdS, Cunha CLF, Pinho ECC, Paiva DdJdS, de Castro NJC, Nascimento VGC, et al. Seroprevalence of Chlamydia trachomatis and Associated Factors among Vulnerable Riverine in the Brazilian Amazon. Int J Environ Res Public Health. 2022;19(23):15969. doi: 10.3390/ijerph192315969 36498044 PMC9736917

[12] Pinho ECC, Galvão JJDS, Martins WM, Goncalves FE, Aben-Athar CYUP, da Silva RAR, et al. Knowledge About Sexually Transmitted Infections and Associated Factors Among Brazilian Riverside People. Nurs Health Sci. 2024;26(4):e70002. doi: 10.1111/nhs.70002 39657781

[13] Gonzalez S, Lopez Velasco PN, Mena Antonio CA, Palazuelos D. Detecting sexually transmitted infections beyond the syndromic approach: lessons from a rural setting in Chiapas, Mexico. Front Reprod Health. 2024;6:1441909. doi: 10.3389/frph.2024.1441909 39114476 PMC11303321

[14] Singh S, Shahid R, Pradhan S. Syndromic diagnosis, sexual behavior, and management in rural population among all cases attending sexually transmitted infection clinic in a tertiary care center from the east-central zone of India: A retrospective study. Indian J Sex Transm Dis AIDS. 2024;45(1):34–8. doi: 10.4103/ijstd.ijstd_34_23 38989097 PMC11233056

[15] Brasil. Ministério da Saúde. Secretaria de Vigilância em Saúde e Ambiente. Boletim Epidemiológico: Sífilis 2024[Internet]. Brasília (DF): Ministério da Saúde; 2025. [cited 2025 Jan 31]. Available from: https://www.gov.br/saude/pt-br/centrais-de-conteudo/publicacoes/boletins/epidemiologicos/especiais/2024/boletim-epidemiologico-de-sifilis-numero-especial-out-2024.pdf

[16] Guimarães AF, Barbosa VLM, Silva MPd, Portugal JKA, Reis MH da S, Gama ASM. Acesso a serviços de saúde por ribeirinhos de um município no interior do estado do Amazonas, Brasil. Revista Pan-Amazônica de Saúde. 2020;11(0). doi: 10.5123/s2176-6223202000178

[17] Machado LFA, Fonseca RRdS, Queiroz MAF, Oliveira-Filho AB, Cayres-Vallinoto IMV, Vallinoto ACR, et al. The Epidemiological Impact of STIs among General and Vulnerable Populations of the Amazon Region of Brazil: 30 years of Surveillance. Viruses. 2021;13(5):855. doi: 10.3390/v13050855 34067165 PMC8151421

[18] Schwartz N, Hunter S, Forbes SM, Gesink D, Hobin E, Anderson LN, et al. The COVID-19 pandemic’s impact on sexually transmitted infections and the modifying role of public health funding: an interrupted time series study. J Public Health (Oxf). 2025;47(3):395–403. doi: 10.1093/pubmed/fdaf053 40349204 PMC12395938

[19] Machado HM, Martins JM, Schörner MA, Gaspar PC, Bigolin A, Ramos MC. National surveillance of Neisseria gonorrhoeae antimicrobial susceptibility and epidemiological data of gonorrhoea patients across Brazil, 2018–2020. JAC Antimicrob Resist. 2022;4(4):dlac076. doi: 10.1093/jacamr/dlac076 PMC9252985 35795244

[20] Vargas S, Calvo G, Qquellon J, Vasquez F, Blondeel K, Ballard R, et al. Point-of-care testing for sexually transmitted infections in low-resource settings. Clin Microbiol Infect. 2022;28(7):946–51. doi: 10.1016/j.cmi.2021.05.052 34118424

[21] Coutinho KD, Valentim RAdM, Vieira GV, Sidrim M, Evangelista PHG, de Oliveira LP. Management Solutions for the Restructuring of Laboratories Associated to the Sentinel Services for Syphilis and Other STIs. Front Public Health. 2022;10:841919. doi: 10.3389/fpubh.2022.841919 35570931 PMC9099240

[22] Miranda AE, Freitas FLS, Passos MRL de, Lopez MAA, Pereira GFM. Public policies on sexually transmitted infections in Brazil. Rev Soc Bras Med Trop. 2021;54(suppl 1):e2020611. doi: 10.1590/0037-8682-611-2020 34008725 PMC8210478

[23] Gottlieb SL, Spielman E, Abu-Raddad L, Aderoba AK, Bachmann LH, Blondeel K, et al. WHO global research priorities for sexually transmitted infections. Lancet Glob Health. 2024;12(9):e1544–51. doi: 10.1016/S2214-109X(24)00266-3 39043199 PMC11342064

[24] World Health Organization. Guidelines for the management of symptomatic sexually transmitted infections[Internet]. Geneva: World Health Organization; 2021 Jun [cited 2025 Jan 31]. Available from: https://www.ncbi.nlm.nih.gov/books/NBK572659/34370424

